# Parapneumonic Effusion Versus Pulmonary Empyema in Children: Analysis of Risk Factors and Laboratory Predictors Through a Single Center Experience

**DOI:** 10.3390/children12081103

**Published:** 2025-08-21

**Authors:** Marta Improta, Francesca Morlino, Roberta Ragucci, Carolina D’Anna, Stefania Muzzica, Vincenzo Tipo, Antonietta Giannattasio, Marco Maglione

**Affiliations:** Pediatric Emergency Department, Santobono-Pausilipon Children’s Hospital, 80129 Naples, Italy; marta.improta@unina.it (M.I.); francesca.morlino@unina.it (F.M.); ro.ragucci@studenti.unina.it (R.R.); c.danna@santobonopausilipon.it (C.D.); s.muzzica@santobonopausilipon.it (S.M.); v.tipo@santobonopausilipon.it (V.T.); a.giannattasio@santobonopausilipon.it (A.G.)

**Keywords:** parapneumonic effusion, pleural empyema, community-acquired pneumonia, pleural drainage

## Abstract

**Background:** Parapneumonic effusion is a common complication of community-acquired pneumonia and can range from a simple inflammatory transudate to an organized purulent collection, known as empyema. Progression to empyema significantly worsens the prognosis, leading to increased morbidity, longer hospital stays, and a greater need for invasive interventions. Several risk factors for pleural effusion and progression to empyema have been identified, but the absence of standardized criteria underline the need for better risk stratification. We analyzed clinical and laboratory data from a cohort of children hospitalized with pneumonia associated with pleural effusion or empyema, to identify predictive risk factors associated with these complications. **Methods**: We retrospectively analyzed clinical and laboratory data from patients admitted to our Pediatric Emergency Department with pneumonia complicated by pleural effusion and compared patients with simple effusion to those with empyema. **Results**: Seventeen children with simple pleural effusion and eighteen with empyema were enrolled. Patients with empyema had higher absolute neutrophil count, higher levels of C-reactive protein, procalcitonin, and ferritin, and lower serum albumin levels. Furthermore, they took a longer time for normalization of inflammatory markers when compared with those with pleural effusion. Invasive interventions, such as pleural drainage, and the need for intensive care were more frequent in the empyema group. **Conclusions**: Pleural effusion and empyema are two common complications of pediatric community-acquired pneumonia. Children developing pleural empyema have higher inflammatory markers and lower levels of serum albumin compared to patients with simple pleural effusion. Morbidity is significantly worse in children with empyema as they are more prone to require invasive interventions and intensive care.

## 1. Introduction

Pleural complications, such as pleural effusion (PEf) and empyema (PEm), are common associated conditions and significant morbidity contributors in pediatric community-acquired pneumonia (CAP). Parapneumonic effusions develop in approximately 0.7–1% of CAP cases, reaching rates as high as 40% among hospitalized children [1]. Even though most effusions do not progress beyond the initial exudative phase and resolve by treating the underlying pneumonia, when the pleural fluid becomes infected and purulent, it evolves into empyema. Despite being associated with low mortality (<3%) and with a complete recovery in most cases, this condition still represents a therapeutic challenge to clinicians, entailing substantial risks for patients as it may require invasive procedures like chest drainage or surgery [2].

*Streptococcus pneumoniae*, *Staphylococcus aureus*, and *Streptococcus pyogenes* are the etiologic agents most frequently associated with PEm, whose incidence has significantly been decreased by the introduction of the pneumococcal conjugate vaccine including an increasing number of serotypes [3,4]. Nevertheless, in recent post-pandemic years, a new increase in the incidence of empyema and other CAP complications has been reported in children [5], possibly reflecting changing serotypes and host–pathogen dynamics.

Given the significantly different burden of simple PEf and PEm, there is a pressing need for early identification of children at high risk for developing PEm by means of biomarkers able to drive more aggressive and timely treatment. The present manuscript aims to analyze clinical and laboratory parameters at the time of hospitalization in a cohort of children with CAP complicated by PEf or PEm in order to assess risk factors for predicting the development of these complications.

## 2. Materials and Methods

### 2.1. Study Design

We conducted a retrospective observational study analyzing pediatric patients admitted to the Pediatric Emergency Department of the Santobono-Pausilipon Children’s Hospital with a diagnosis of pneumonia complicated by pleural effusion between August 2024 and May 2025. Inclusion criteria were age below 14 years and a diagnosis of CAP complicated by pleural effusion as confirmed by means of chest X-ray, lung ultrasound, and chest computed tomography (CT), alone or in combination. The enrolled patients were divided into two distinct groups defined on established radiological criteria [6]: children with pleural empyema (PEm group) and those with simple parapneumonic effusion (PEf group). PEf was defined as clear anechoic or cloudy hypoechoic fluid with or without swirling particles, whereas PEm was diagnosed when lung ultrasound showed the presence of hypoechoic fluid partitioned by fibrin strands, sometimes with a septated or multi-loculated appearance and/or no clear demarcation between the lung and pleural components [6]. Therefore, as patients’ classification was primarily based on lung ultrasound findings, this imaging technique had a central role in our study and was crucial in both assessment and monitoring of pleural involvement. As with chest X-ray and CT scans, all lung ultrasound studies were performed and interpreted by experienced pediatric radiologists.

### 2.2. Data Collection

At admission, presenting symptoms and vital parameters, including oxygen saturation and respiratory rate, were recorded. Laboratory assessment included complete blood cell count, inflammatory markers including C-reactive protein (CRP), procalcitonin (PCT), and ferritin. Additionally, complete biochemical evaluation including serum albumin and electrolytes levels was performed. When available, serial laboratory assessment during the hospital stay was used to define the time required for CRP normalization, defined as a CRP value < 3 mg/L, and for PCT normalization, defined as PCT < 0.25 ng/mL.

The microbiological profile of the recruited patients was defined by means of urinary pneumococcal antigen detection, molecular biology and culture test on respiratory samples, blood samples or pleural fluid (when available in patients undergoing chest drainage), and *Mycoplasma pneumoniae* serology. This comprehensive microbiological assessment, aimed at identifying possible responsible pathogens and at guiding antibiotic therapy, is part of the routine diagnostic management of complicated pneumonia at our center.

### 2.3. Statistical Analysis

Categorical variables are shown as numbers and percentages, while continuous variables are shown as median value and range. Comparisons between PEm and PEf groups were performed using Fisher’s exact test for categorical variables, whereas the Mann–Whitney U test was used for continuous variables. A two tailed *p*-value < 0.05 was considered statistically significant. Multivariate logistic regression models were developed to assess independent variables associated with the pleural complication (PEf group versus PEm group). The independent variables examined were white blood cells (WBCs), neutrophil and lymphocyte count, CRP, PCT, ferritin and albumin. For all these variables, sensitivity and specificity tests were conducted and the Area Under the Curve (AUC) was calculated using the Receiver Operating Characteristic (ROC) curve to assess the diagnostic efficacy of these indicators in identifying PEf or PEm. The AUC values were then interpreted based on predefined criteria, categorized as fail (0.5–0.6), poor (0.6–0.7), fair (0.7–0.8), good (0.8–0.9), or excellent accuracy (0.9–1.0) [7]. The optimal cut-off points were determined by choosing the points that maximized the Youden J statistic (sensitivity + specificity − 1) [8]. Statistical analysis was performed using StataCorp LLC Stata 17.0 (College Station, TX, USA).

## 3. Results

### 3.1. Patient Characteristics and Clinical Presentation

**Figure 1:**
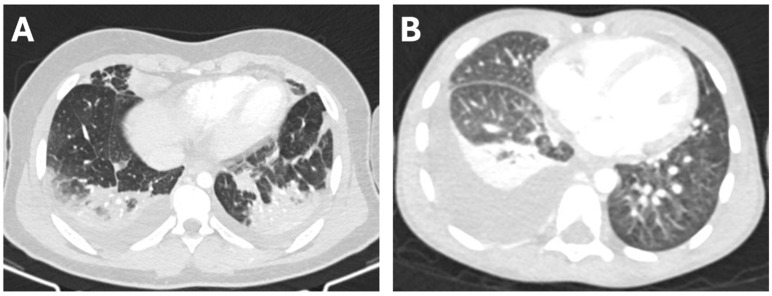
Transverse chest CT scans from a 13-year-old boy with PEf associated with parainfluenza virus pneumonia (**A**), and from a 5-year-old boy with PEm associated with pneumococcal pneumonia (**B**).

We enrolled 35 patients with pneumonia complicated by pleural effusion, of whom 18 developed PEm, whereas 17 had simple PEf. Chest CT scans from two cases are reported in Figure 1. No differences in gender distribution or age were found between the two groups (Table 1).

Comorbidities were rare in both groups. Only one patient in the PEf group had pre-existing bronchial asthma, whereas one case of atrial septal defect was reported in the PEm group. No patients in the PEm group had respiratory comorbidities. Most patients were up-to-date with the pneumococcal vaccinations as they had already received all the scheduled doses of 13-valent pneumococcal conjugate vaccination according to the age at the time of admission. Five patients (three belonging to the PEf and two to the PEm groups, respectively) had not received all the scheduled antipneumococcal doses, whereas two children, one with PEf and one with PEm, respectively, were completely unvaccinated for *Streptococcus Pneumoniae*, due to vaccination hesitancy.

Clinical presentation was remarkably similar between the two groups, with fever being the predominant symptom in nearly all patients. Although statistical significance was not achieved, a trend towards a higher prevalence of dyspnea and abdominal or chest pain was observed in the PEm group. Similarly, no significant differences between the two groups were observed when comparing vital parameters at presentation. Particularly, oxygen saturation and respiratory rate were similar between the groups (Table 1).

No patient with PEf required intensive care or invasive procedures. In contrast, within the PEm group, four subjects required intensive care unit admission (in two cases with invasive respiratory support), and most patients needed strict monitoring of vital parameters in the sub-intensive care unit, and/or pleural drainage. In three patients, thoracic surgery was needed due to complications (lobectomy due to parenchymal necrosis in two cases, pleural debridement in one case). Consequently, we found a significantly longer duration of hospital stay in the PEm group than in the PEf group (Table 1).

### 3.2. Laboratory Findings

In contrast to clinical and demographic features, laboratory parameters revealed statistically significant differences between the two groups (Table 2).

First, a relevant difference was observed in white blood cell count, with patients with PEm showing a more pronounced neutrophil leukocytosis when compared to the PEf group. Differences between the two groups were even more evident when comparing the inflammatory markers at admission, with CRP, PCT and ferritin levels significantly more elevated in PEm than in PEf patients. Furthermore, despite follow-up laboratory data being available for 30 patients regarding CRP (18 with PEm and 12 with PEf) and 32 patients regarding PCT (18 with PEm and 14 with PEf), time to CRP and PCT normalization resulted significantly longer in PEm patients. Finally, serum albumin levels at admission were significantly lower in children with PEm than in those with PEf.

**Figure 2:**
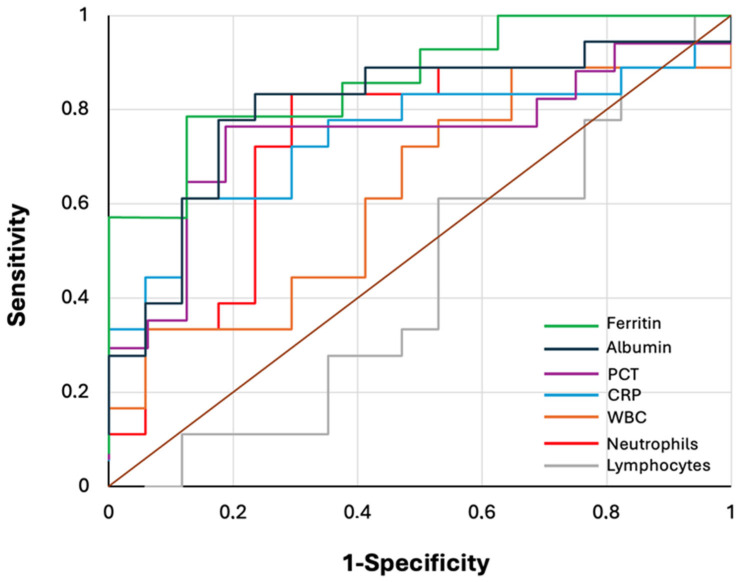
ROC curves of laboratory parameters at presentation for discriminating between PEm and PEf.

Table 3 and Figure 2 summarize ROC curve analysis results of seven laboratory parameters assessed for discriminating between PEf and PEm. ROC curve analysis demonstrated that five out of the seven tested parameters showed statistically significant ability to discriminate between the two conditions. Neutrophil count, CRP, PCT, ferritin, and albumin all achieved AUC values above 0.72, indicating fair-to-good diagnostic accuracy. Ferritin showed the highest overall performance and, with a cut-off value of 472.8 ng/mL, sensitivity and specificity were 79% and 87%, respectively. White blood cell and lymphocyte count did not reach statistical significance.

The statistically significant results observed in the univariate analysis were not confirmed by the multivariate analysis of the single laboratory parameters (Table 4). The only parameter whose odds ratio suggested a role in discriminating between PEm and PEf, serum albumin, revealed to be largely imprecise and, as other variables, did not achieve statistical significance.

Overall, microbiological investigations allowed for an etiological diagnosis in 26 patients, with positive molecular biology on respiratory samples as only etiological support in eight more children (Table 5). The most frequently detected pathogen was *Mycoplasma pneumoniae*. According to positive serology and/or pathogen detection on respiratory samples, 10 out of 17 infections in the PEf group and 7 out of 18 in the PEm group were sustained by *Mycoplasma pneumoniae*, sometimes in co-infection with other bacterial or viral pathogens. No etiological diagnosis was achieved in one child, whose microbiological investigations were all inconclusive.

## 4. Discussion

The present study compared clinical and laboratory data from two groups of children hospitalized because of CAP complicated by PEf and PEm, respectively. Our main finding was the lack of clinical data at admission that could adequately distinguish the two groups. On the contrary, several laboratory biomarkers, namely, neutrophil leukocytosis, high CRP, procalcitonin, ferritin and low serum albumin levels were significantly related to PEm, thus suggesting a more intense systemic inflammatory response in these patients.

These findings were further strengthened by the univariate ROC analysis, that showed, for five out of seven laboratory parameters, a statistically significant discriminative ability between patients with PEm and those with PEf, with AUC values exceeding 0.72. These results indicate that, when assessed individually, these parameters have a relevant capacity to discriminate between the two diagnostic groups, and the identified cut-off values, once confirmed on larger populations, may have relevant clinical implications on the diagnostic approach to these conditions.

Unfortunately, in the multivariate logistic regression model, none of the parameters retained statistical significance, and odds ratios were close to 1 with narrow confidence intervals. This apparent discrepancy is likely explained by collinearity among predictors, as most tested variables reflect overlapping biological processes, namely, systemic inflammation. When such correlated parameters are included in the same model, their independent contribution to the outcome is substantially reduced. In addition, the small sample size has likely limited the statistical power of the multivariate analysis, particularly given the number of predictors considered. While ROC analysis optimizes cut-off points and evaluates each parameter individually, logistic regression assesses their independent predictive value, penalizing redundancy.

Taken together, these findings suggest that, although multiple laboratory parameters can individually discriminate between PEm and PEf, none retained statistical significance in the multivariate model, likely due to overlapping information and limited sample size, that is a major limit of our study. This prevents from drawing robust conclusions and makes it mandatory to verify the correlations we found in larger studies to make them reliable and generalizable.

Comparing our results with previously published data on the topic is only partially feasible as, in most studies, risk factors and predictive biomarkers for the development of PEf and PEm have been assessed in comparison to uncomplicated CAP [9,10,11]. Furthermore, most studies considered all parapneumonic pleural fluid collections as a single entity within the wide group of “complicated CAP”; very different conditions were also included such as necrotizing pneumonia and lung abscess. Conversely, we did not include in our cohort patients with uncomplicated CAP, and focused on two specific complications, one of which, PEf, is generally more easily managed and entails less morbidity than the other. Even though these strict selection criteria have limited our sample size, they certainly represent a strength of our analysis. Nevertheless, the association we found between higher inflammatory markers, particularly CRP, and PEm has been reported in several studies considering PEm together with other CAP complications [9,10,11]. Interestingly, a case-control study from India identified six predictors of complicated PEf/PEm that were ultimately joined in a score with a reported sensitivity and specificity of 80% and 94%, respectively [10]. Of these predictors, namely, pre-hospitalization ibuprofen intake, infective focus elsewhere, hypoalbuminemia < 3.1 g/dL, serum CRP > 20 mg/dL, Hb < 10 g/dL and total leucocyte count > 10,000 cells/μL, at least three were in line with our univariate analysis. Of note, we did not record data regarding pre-hospitalization treatments such as ibuprofen, but clinicians’ awareness regarding the potential threat represented by non-steroidal anti-inflammatory drug abuse in children with acute infections is increasing in our setting [12]. Although still debated due to the poor quality of the available studies [13], the correlation between ibuprofen treatment and CAP complications highlighted in several reports [11,14,15] suggests caution in the use of this drug in affected children.

With regards to the clinical parameters at admission, previous studies have reported that high respiratory rate [16], high fever [9] and chest pain [11] are associated with complicated CAP. Noteworthy, and in line with these data, our analysis showed a trend towards a higher prevalence of dyspnea, abdominal and chest pain in children with PEm, even though, likely due to the small sample size, statistical significance was not achieved. Further predictors of severity and of longer hospitalizations in complicated CAP have been proposed, namely, higher levels of lactate dehydrogenase (LDH) and lower levels of glucose in the pleural fluid [17]. Nevertheless, their applicability is limited, as it depends on invasive procedures to collect and characterize pleural fluid composition, that are fortunately not always required in children with simple parapneumonic pleural collections.

The search for predictive factors and the definition of diagnostic and therapeutic recommendations of PEm and other CAP complications represent pressing needs for clinicians, as difficulties and doubts in the management of these conditions are experienced daily in pediatric wards. The uncertainty regarding the underlying etiology, that is often derived from the combination of serology, microbiological and molecular tests on different biological samples, leads to heterogeneous antibiotic treatments that deeply rely on the experience of the single center. Furthermore, the multidisciplinary management of pleural collections, often shared with pediatric surgeons and radiologists, makes a uniform approach difficult to achieve, particularly in borderline cases, when immediate chest drainage positioning and watchful monitoring during medical treatment represent alternative options. At present, management of pediatric pleural disease relies on largely widespread guidelines released by the British Thoracic Society 20 years ago [18]. Despite its high quality and validity even after two decades, this document is limited by the lack of updated data on the epidemiology of pediatric respiratory pathogens, particularly as a result of the new antipneumococcal vaccination strategies [4,19] and of the deep changes observed in the post-pandemic years [20,21,22]. Finally, as recent evidence regarding diagnostic tools [23,24,25] and therapeutic approaches [26,27,28,29] to pediatric pleural disease deserve adequate evaluation, new guidelines on this topic are highly desirable. Recently, the German Association of Scientific Medical Societies has partially filled this gap with the publication of the guidelines for pediatric CAP management, including several useful indications regarding diagnosis and treatment of parapneumonic effusion and empyema [30]. This document has the relevant merit to provide updated recommendations regarding PEf and PEm management, including indications on pleural puncture and chest tube placement, intrapleural fibrinolysis and epidemiology-based antibiotic choice. Furthermore, it correctly underlines the central role of lung ultrasound as a necessary diagnostic and monitoring tool for pleural collections. Nevertheless, the publication of this document solely in German, along with the fact that it is not indexed in the main scientific literature databases, has limited its dissemination, that deserves to be implemented within the pediatric community also beyond German borders.

Treatment of complicated and uncomplicated parapneumonic effusions generally moves from parenteral antibiotics, often with combinations of different drugs, even though the effectiveness of such approach has been questioned [27]. Further interventions, primarily indicated in frank empyema with poor response to antibiotic therapy, or in large pleural collections, include chest drainage both for allowing adequate parenchymal re-expansion and for administration of intrapleural fibrinolytics [31]. In line with current literature [25,26], also in our practice three daily doses of intrapleural urokinase have proven useful in helping the resolution of PEm, particularly when ultrasound characteristics suggest organized or multiloculated fluid collections.

Our study has several limitations. First, the small sample size weakened our analysis, particularly when laboratory parameters were assessed within a multivariate model, preventing the achievement of statistical significance in the evaluation of diagnostic accuracy of some parameters. Therefore, the cut-off values identified for each laboratory parameter should be interpreted with caution until further validation, as the diagnostic power emerged from our data is limited. Furthermore, despite the fact that patients with CAP are managed with a relatively standardized diagnostic protocol at our center, the retrospective nature of our study entailed that, except for some procedures (e.g., laboratory parameters and lung ultrasound at admission), not all subjects underwent the same radiologic procedures, and in some of them, follow-up laboratory data were not collected.

## 5. Conclusions

Our findings support the lack of specific clinical parameters at the time of hospital admission, which allows for a reliable distinction between children with simple parapneumonic effusion and those with pleural empyema. Nevertheless, neutrophil leukocytosis, elevated inflammatory markers, and low serum albumin levels represent useful markers that should raise clinicians’ suspicion for purulent pleural effusion in children with complicated CAP. Validation of our findings in larger populations and further research are strongly needed to better define biomarkers or risk factors that could help with a timely diagnosis of PEm. Indeed, early antibiotic treatment and careful timing of chest drainage may warrant a better prognosis with reduced need for surgical interventions.

## Figures and Tables

**Table 1:** Demographic data, clinical characteristics, and therapeutic interventions from patients with simple pleural effusion (PEf group) and pleural empyema (PEm group).

	PEf Group (n = 17)	PEm Group (n = 18)	*p*-Value
**Demographic data**			
Male sex, n (%)	8 (47.1)	8 (44.4)	1.0
Female sex, n (%)	9 (52.9)	10 (55.6)	1.0
Age (yrs) *	7.1 (1.4–13.4)	5.2 (0.2–13)	0.34
**Comorbidity**			
Respiratory conditions, n (%)	1 (5.8)	0 (0)	1.0
Other, n (%)	0 (0)	1 (5.5)	1.0
**Clinical Features**			
Fever, n (%)	16 (94.1)	17 (94.4)	1.0
Dyspnea, n (%)	8 (47.1)	12 (66.6)	0.31
Abdominal Pain, n (%)	3 (17.6)	8 (44.4)	0.15
Chest Pain, n (%)	1 (5.9)	3 (16.7)	0.61
Cough, n (%)	13 (76.5)	11 (61.1)	0.47
SpO_2_% ^1^, median (range)	93.8 (85–100)	93.2 (85–99)	0.3
RR ^2^ (acts/min), median (range)	30 (20–70)	30 (25–66)	0.3
**Therapeutic Interventions**			
Invasive respiratory support, n (%)	0 (0)	2 (11.1)	0.48
Pleural Drainage, n (%)	0 (0)	13 (72)	<0.001
Surgery, n (%)	0 (0)	3 (16.6)	0.23
Sub-Intensive Care admission, n (%)	1 (5.9)	12 (66.6)	<0.001
Intensive Care Unit admission, n (%)	0 (0)	4 (22.2)	0.1
Hospital stay (days) *	3 (2–7)	23 (7–88)	<0.001

^1^ Oxygen saturation; ^2^ Respiratory rate; * Median and ranges.

**Table 2:** Laboratory data from patients with simple pleural effusion (PEf group) and pleural empyema (PEm group).

Laboratory Data	PEf Group (n = 17)	PEm Group (n = 18)	*p*-Value
WBC ^1^ (cell/μL)	11,050 (6810–28,810)	22,355 (9420–38,300)	<0.001
Neutrophils (cell/μL)	6660 (4450–26,660)	10,020 (290–30,870)	0.05
Lymphocytes (cell/μL)	2530 (830–8410)	1580 (870–7660)	0.10
CRP ^2^ (mg/L)	30.34 (2.23–371.99)	273.9 (31–578.79)	<0.001
PCT ^3^ (ng/mL)	0.25 (0.05–15.0)	4.6 (0.07–175)	<0.01
Ferritin (ng/mL)	297.45 (26.5–514)	691.7 (312–2375)	0.001
Albumin (g/dL)	4 (2.9–4.8)	3.1 (2.5–5.0)	0.001
Time to CRP normalization * (days)	3 (0.0–6.0)	15 (3–61)	<0.001
Time to PCT normalization * (days)	0 (0–6)	10 (0–25)	<0.001

Data are expressed as median and ranges. ^1^ White blood cells; ^2^ C-reactive protein; ^3^ Procalcitonin; * Data available for 30/35 patients for CRP and 32/35 patients for PCT.

**Table 3:** ROC curve analysis results of laboratory parameters for discriminating between PEm and PEf.

	Cut-Off Value	AUC ^1^ (95%CI)	*p*-Value	Sensitivity (%)	Specificity (%)
WBC ^2^	20,560	0.624 (0.438–0.810)	0.19	33.3	94.1
Neutrophils	7450	0.727 (0.559–0.895)	0.008	83	71
Lymphocytes	1470	0.431 (0.239–0.623)	0.48	61	47
CRP ^3^	185	0.735 (0.568–0.902)	0.006	61	88
PCT ^4^	0.6	0.746 (0.577–0.915)	0.004	76	81
Ferritin	472.8	0.835 (0.665–1)	<0.001	79	87
Albumin	3.7	0.822 (0.681–0.963)	<0.001	83	76

^1^ Area under the curve; ^2^ White blood cells; ^3^ C-reactive protein; ^4^ Procalcitonin.

**Table 4:** Multivariate analysis of laboratory parameters.

	OR ^1^	95% CI		*p*-Value
WBC ^2^	0.99	0.99	1.00	0.52
Neutrophils	1.01	0.99	1.00	0.54
Lymphocytes	1.01	0.99	1.00	0.51
CRP ^3^	0.99	0.97	1.02	0.74
PCT ^4^	0.98	0.83	1.15	0.79
Ferritin	0.99	0.99	1.00	0.17
Albumin	2.39	0.19	30.23	0.50

^1^ Odds ratio; ^2^ White blood cells; ^3^ C-reactive protein; ^4^ Procalcitonin.

**Table 5:** Microbiological data from patients with simple pleural effusion (PEf group) and pleural empyema (PEm group).

	PEf Group (n = 17)	PEm Group (n = 18)	*p*-Value
**Microbiological Data**			
Urinary Pneumococcal Antigen (n/tested, %)	0/11 (0)	4/16 (25)	0.10
Positive Mycoplasma Serology (n/tested, %)	8/14 (57.1)	7/13 (53.8)	1.0
Positive Blood Culture (n/tested, %)	1/12 (8.3)	2/16 (12.5)	1
Isolated pathogen on respiratory sample, n (%) *			
* Mycoplasma pneumoniae*	7 (41.2)	1 (5.5)	
* Chlamydia pneumoniae*	0 (0)	1 (5.5)	
Metapneumovirus	2 (11.8)	1 (5.5)	
Adenovirus	2 (11.8)	3 (16.7)	
Respiratory syncytial virus	2 (11.8)	1 (5.5)	
Rhino/Enterovirus	4 (23.5)	6 (33.3)	
Parainfluenza virus	4 (23.5)	2 (11.1)	
Influenza virus	0 (0)	1 (5.5)	
Coronavirus	2 (11.8)	1 (5.5)	
None	4 (23.5)	2 (11.1)	

* Nucleic acids identified by polymerase chain reaction testing. Total exceeds 100% due to frequent detection of multiple pathogens.

## Data Availability

The original contributions presented in this study are included in the article. Further inquiries can be directed to the corresponding author.

## Abbreviations

The following abbreviations are used in this manuscript:

CAP	Community Acquired Pneumonia
PEf	Pleural Effusion
PEm	Pleural Empyema
CT	Computed Tomography
WBC	White Blood Cells
CRP	C-Reactive Protein
PCT	Procalcitonin
SpO_2_	Peripheral Oxygen Saturation
LDH	Lactate Dehydrogenase
ROC	Receiver Operating Characteristic
AUC	Area Under the Curve
